# Metabolic Reprogramming, Gut Dysbiosis, and Nutrition Intervention in Canine Heart Disease

**DOI:** 10.3389/fvets.2022.791754

**Published:** 2022-02-15

**Authors:** Qinghong Li

**Affiliations:** Nestlé Purina Research, Saint Louis, MO, United States

**Keywords:** mitral valve disease (MVD), energy metabolism, amino acids, nutrition, disease, heart failure, microbiome, cardiac metabolism

## Abstract

This review provides a state-of-the-art overview on recent advances in systems biology in canine cardiac disease, with a focus on our current understanding of bioenergetics and amino acid metabolism in myxomatous mitral valve disease (MMVD). Cross-species comparison is drawn to highlight the similarities between human and canine heart diseases. The adult mammalian heart exhibits a remarkable metabolic flexibility and shifts its energy substrate preference according to different physiological and pathological conditions. The failing heart suffers up to 40% ATP deficit and is compared to an engine running out of fuel. Bioenergetics and metabolic readaptations are among the major research topics in cardiac research today. Myocardial energy metabolism consists of three interconnected components: substrate utilization, oxidative phosphorylation, and ATP transport and utilization. Any disruption or uncoupling of these processes can result in deranged energy metabolism leading to heart failure (HF). The review describes the changes occurring in each of the three components of energy metabolism in MMVD and HF. It also provides an overview on the changes in circulating and myocardial glutathione, taurine, carnitines, branched-chain amino acid catabolism and tryptophan metabolic pathways. In addition, the review summarizes the potential role of the gut microbiome in MMVD and HF. As our knowledge and understanding in these molecular and metabolic processes increase, it becomes possible to use nutrition to address these changes and to slow the progression of the common heart diseases in dogs.

## Introduction

The adult mammalian heart has a very high demand for energy in order to sustain its constant contractile activities and meet its basal metabolic needs (1). More than 70% of ATPs in the normal adult heart are produced by fatty acid oxidation (FAO) in the complex mitochondrial machinery while the remaining balance comes from the oxidation of other substrates including glucose (1). The heart is metabolically flexible and shifts its preference in energy substrates in accordance with different developmental stages, physiological, or pathological conditions (2). The concept of the failing heart as an energy starved engine that runs out of fuel was initially proposed by Herrmann and Decherd almost one century ago and continues to attract considerable research interests today (1–7). The failing heart can exhibit an energy deficit of up to 40% less ATP than a healthy heart (5, 8), increasing its reliance on glucose and other energy substrates as fuel in the context of reduced capacity of FAO (1, 2, 9). Inside the cardiac myocytes, glucose is either converted to sorbitol by the polyol pathway or phosphorylated by hexokinase to glucose 6-phosphate, which subsequently goes through several metabolic pathways including glycolysis (10). Recently, a growing body of evidence indicates ketone bodies as a significant fuel source in the failing and diseased heart (7, 11–14). Pathological alternations of these energy metabolic pathways are associated with impaired signal transductions and altered energy and redox homeostasis leading to contractile dysfunction. Although the pathophysiology of heart failure (HF) is complex and multifactorial (15), strategies that aim to improve cardiac energy metabolism, as an example, by switching to a more efficient myocardial energy substrate, have begun to show promise (7, 13, 16–19).

The concentrations of myocardial and circulating amino acids change in the failing heart in humans and animal models (20–22). Total free amino acids were increased in the humans failing right ventricles (23). Branched-chain amino acid (BCAA) catabolic deficiency is associated with the failing heart in humans and animal models (24–27). Several uremic toxins, many of which are amino acid metabolic products, are associated with heart disease (22, 28). However, the contribution of amino acid metabolic reprogramming to cardiac health and disease has been understudied and underappreciated. In addition, several gut microbiota-produced metabolites have been associated with the cardiovascular disease although no causal relationship has been established (29–31). Myxomatous mitral valve disease (MMVD), the most common naturally occurring heart disease in dogs, is characterized as a slow progressive MV degeneration, which causes mitral regurgitation and, in some cases, can lead to congestive heart failure (CHF) (13, 32, 33). Canine MMVD is very similar to the primary MV prolapse in humans at the morphological, pathophysiological, and molecular levels, and is considered as a model for MV prolapse (34–37). The TGF-β and serotonin (5-HT) signaling pathways have been implicated in the physiopathogenesis of MMVD in both humans and dogs. The observations included increased valvular expressions in genes and proteins in both pathways, increased 5-HT concentrations in circulation, myocardial and valvular tissues in dogs with MMVD. The comparative pathophysiology and the underlying signaling mechanisms by TGF-β and 5-HT have been extensively reported and reviewed (36–42). This review will summarize current advances in cardiac energy and amino acid metabolic reprogramming, associations between gut dysbiosis and heart disease, and opportunities for nutritional intervention.

## Cardiac Energy Metabolism

**Figure 1:**
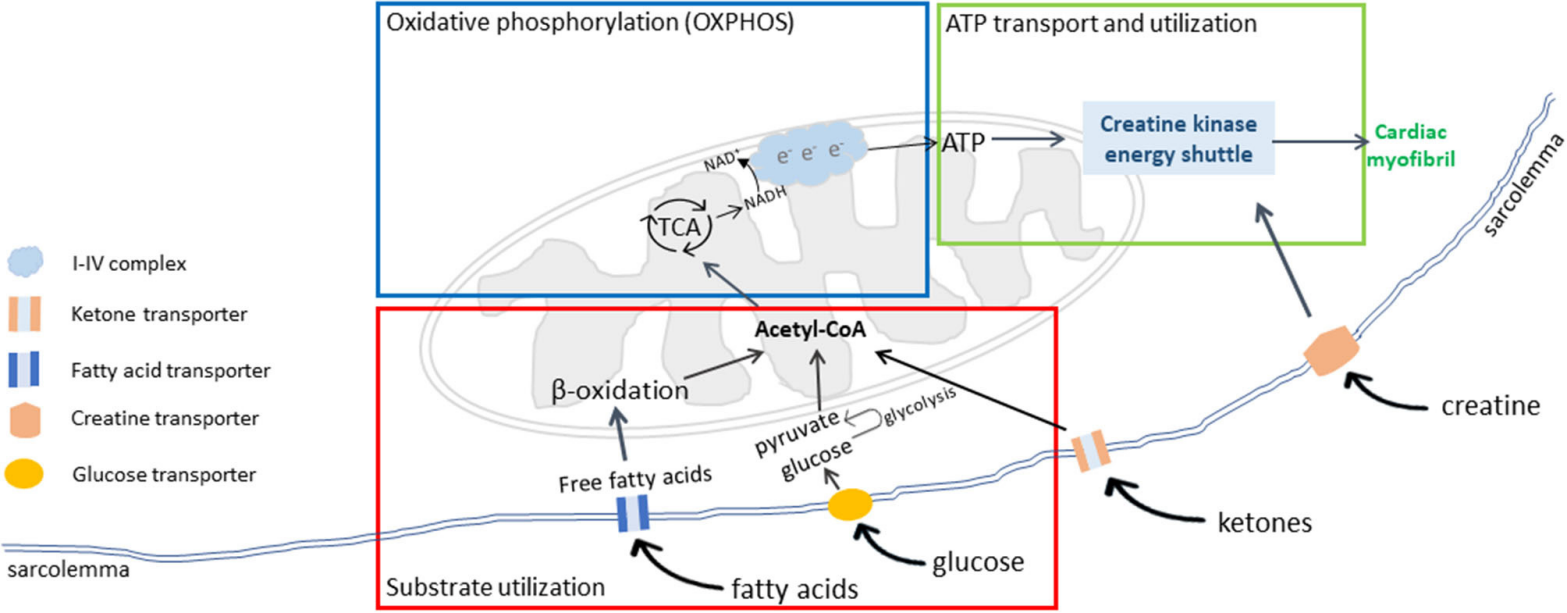
Cardiac energy metabolism. There are three interconnected components of cardiac energy metabolism: substrate transport and utilization (red box), mitochondrial oxidative phosphorylation (OXPHOS) for energy production (blue box), and ATP transport and utilization by cardiac myofibrils (green box). Adapted from Neubauer (6).

The failing heart undergoes extensive metabolic remodeling (43, 44). Cardiac energy metabolism is composed of three interconnected components: substrate utilization and transfer, ATP production by oxidative phosphorylation (OXPHOS), and ATP transfer and utilization by myofibrils (Figure 1). Disruptions or uncoupling of these components may cause derangements in cardiac energy metabolism. This review will describe changes in each of the three components in the failing heart and MMVD in dogs.

### Energy Substrate Utilization

#### Fatty Acid Utilization

**Figure 2:**
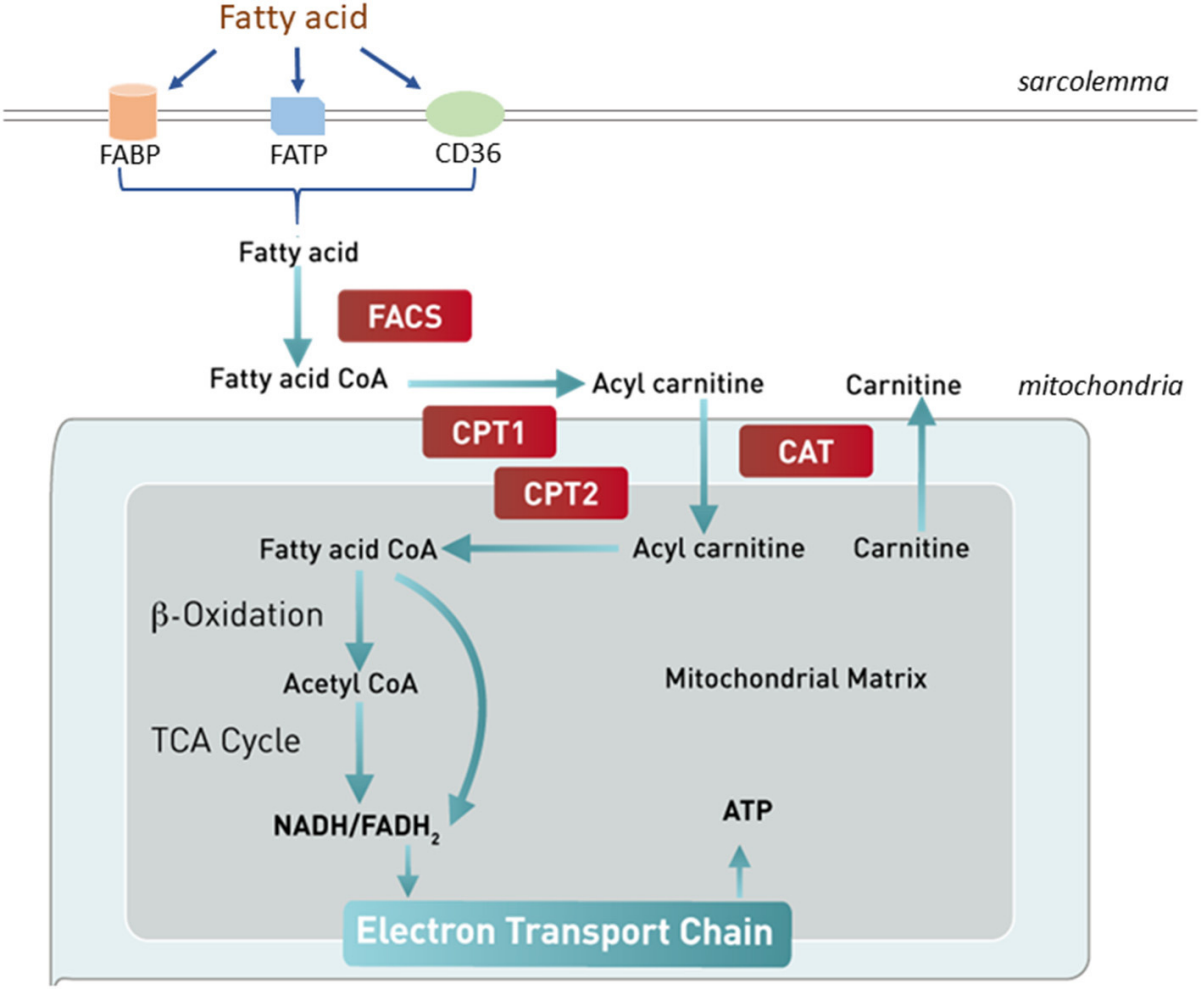
Fatty acid uptake and oxidation. Free fatty acids in circulation enter the cardiomyocytes through either passive diffusion or a carrier protein-facilitated pathway, which involves fatty acid binding protein (FABP), fatty acid translocase (CD36/FAT), or fatty acid transporter protein (FATP). Cytosolic fatty acids are esterified to become fatty acyl CoA by fatty acyl CoA synthase (FACS). The acyl group of fatty acyl-CoA is transferred to carnitine to form acylcarnitine by carnitine palmitoyltransferase 1 (CPT1). The acylcarnitine enters the mitochondrial inner matrix *via* the carnitine shuttle and is converted to fatty acyl CoA by CPT2. The fatty acyl CoA goes through several cycles of β-oxidation producing NADH, FADH_2_, and acetyl CoA, which enters the TCA cycle for ATP production. CAT, carnitine translocase.

In a normal mammalian heart, 70–90% of energy requirement comes from FAO, while the remaining balance comes from glycolysis and oxidation of lactate, and to a small degree, from ketolysis and amino acid oxidation (44, 45). However, the relative contribution of each substrate to the cardiac energy production can vary greatly depending on substrate availability, metabolic demand, and cardiac health condition (44). In the early phase of HF, minor reductions in fatty acid uptake and oxidation are observed, while significant decreases in FAO are detected in advanced HF (6, 45–47). Circulating free fatty acids (FAs) cross the sarcolemmal membrane either through passive diffusion or a carrier protein-assisted pathway (Figure 2). These protein carriers include FA binding protein (FABP), FA transporter protein (FATP), and FA translocase (CD36/FAT). Cytosolic FAs are esterified to become fatty acyl CoA, from which its acyl group is transferred to carnitine to form acylcarnitine by carnitine palmitoyltransferase 1 (CPT1). The acylcarnitine enters the mitochondrial inner matrix *via* the carnitine shuttle and is converted to fatty acyl CoA by CPT2. The fatty acyl CoA goes through several cycles of β-oxidation producing the reduced forms of both nicotinamide adenine dinucleotide (NADH) and flavin adenine dinucleotide (FADH_2_), and acetyl CoA, which enters the TCA cycle for ATP production. The complex regulation of FAO pathway occurs at essentially every step, including the availabilities of circulating free fatty acids, fatty acid uptake and transport across cardiac sarcolemma, fatty acid esterification to become fatty acyl-CoA esters, mitochondrial update *via* the carnitine shuttle, and sequential β-oxidations of long-chain acyl-CoA into acetyl-CoA, and biochemical reactions in the TCA cycle and electron transport chain (ETC) (1).

In dogs with MMVD, fatty acid uptake and transport to cytoplasm and fatty acid conversion to fatty acyl-CoA esters are altered (48). In an RNA-seq transcriptomics study, fatty acid binding protein was downregulated in the MV of preclinical MMVD dogs compared with non-MMVD dogs (48). In addition, long-chain acyl-CoA synthetase, the enzyme that converts long-chain fatty acids to acyl CoA esters, was downregulated in both the left ventricle (LV) and MV (48). These changes suggest impairments in the fatty acid transport and utilization pathway that may lead to deranged bioenergetics.

#### Glucose Utilization

**Figure 3:**
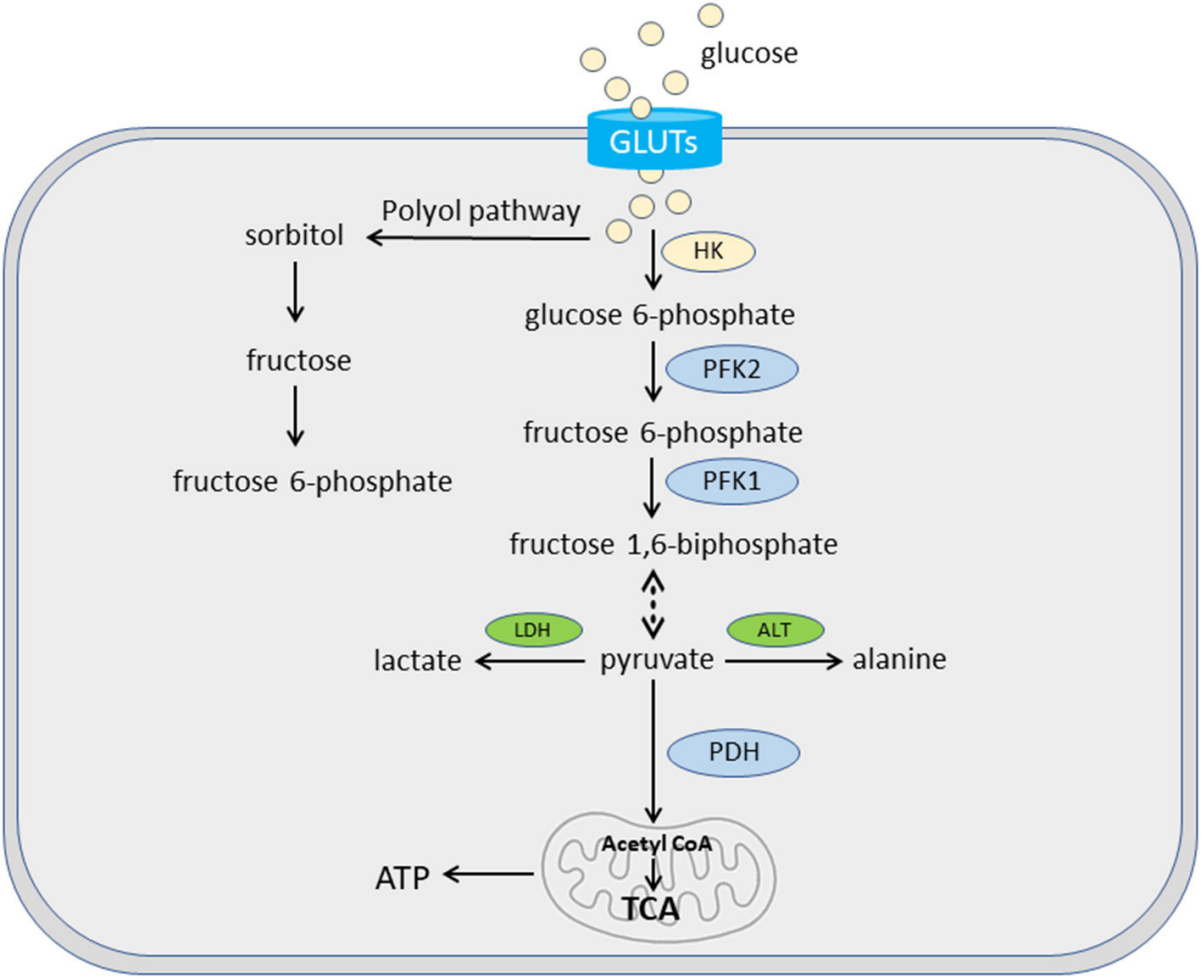
Glucose uptake and oxidation. Glycolysis plays an important role in cardiac bioenergetics. In cardiomyocytes, glucose is transported *via* glucose transporters (GLUTs). Some glucose is converted to fructose and then fructose 6-phosphate for glycolysis *via* polyol pathway. The majority of glucose goes through a series of enzymatic reactions of glycolysis to be converted to pyruvate. These enzymes include hexokinase (HK) for glucose 6-phosphate, phosphofructokinase 2 (PFK2) for fructose 6-phosphate, and PFK1 for fructose 1,6-biphosphate. Pyruvate can be reduced to lactate by lactate dehydrogenase (LDH) in cytosol or oxidized to acetyl-CoA by pyruvate dehydrogenase (PDH) in the mitochondria. A small amount of pyruvate can be converted into alanine by alanine transaminase (ALT).

In cardiac hypertrophy, there is a significant metabolic shift from FAO to glucose (10, 43). Glucose oxidation is more oxygen efficient than FAO, but produces less ATP per molecule. The complete oxidation of 1 palmitate (C16:0) molecule generates 105 ATP molecules, and consumes 46 oxygen atoms, whereas the complete oxidation of 1 glucose molecule generates 31 ATP molecules and consumes only 12 oxygen atoms. The fluxes of glucose and fatty acids are regulated by a feedback mechanism known as the Randle cycle or the glucose-fatty acid cycle (49), which involves the competition between glucose and fatty acids for oxidation. In cardiomyocytes, the majority of glucose is metabolized through glycolysis, which produces pyruvate and ATP. Pyruvate can be reduced to lactate by lactate dehydrogenase in cytosol or oxidized to acetyl-CoA by pyruvate dehydrogenase to fuel the TCA cycle in mitochondria (10) (Figure 3). During hypertrophied growth and remodeling, FAO is decreased with a concomitant increase in glucose utilization (6, 10). However, decreased glucose oxidation was also reported in the development of HF (50). Glucose enters mammalian cells *via* facilitated diffusion, a process regulated by transmembrane glucose transporters (GLUTs) (51). Both GLUT1 and GLUT4 have a well-established role in myocardium. GLUT1 is abundant in the fetal heart whereas GLUT4 is the predominant isoform in the adult heart (10). In dogs with preclinical MMVD, transcriptional changes in GLUT3 and GLUT6 were reported: increased expression of GLUT3 was observed in both the LV and MV, while GLUT6 expression was upregulated in the MV (48). No change was found in either GLUT1 or GLUT4. One possibility is that dogs use different GLUT isoforms than humans or rodents. Interspecies expression difference in GLUT was reported. For example, human β-cells predominantly express GLUT1 while its expression of GLUT2 is 100-fold lower than in rat β-cells (52). Nevertheless, the study did not rule out the possible involvement of other GLUT isoforms in myocardial glucose utilization in dogs. GLUT3, a high-affinity GLUT isoform and a major glucose transporter for the brain, is also present in human adult and fetal myocardium (53, 54). GLUT6 knockout mutant mice show little metabolic effect (55), suggesting a redundant role of GLUT6 in the murine heart. In an untargeted serum metabolomics study, circulating glucose concentration was lower while lactate level was higher in preclinical MMVD dogs vs. non-MMVD dogs (48). The data supported the hypothesis of increased glucose utilization in dogs with MMVD.

#### Ketone Utilization

**Figure 4:**
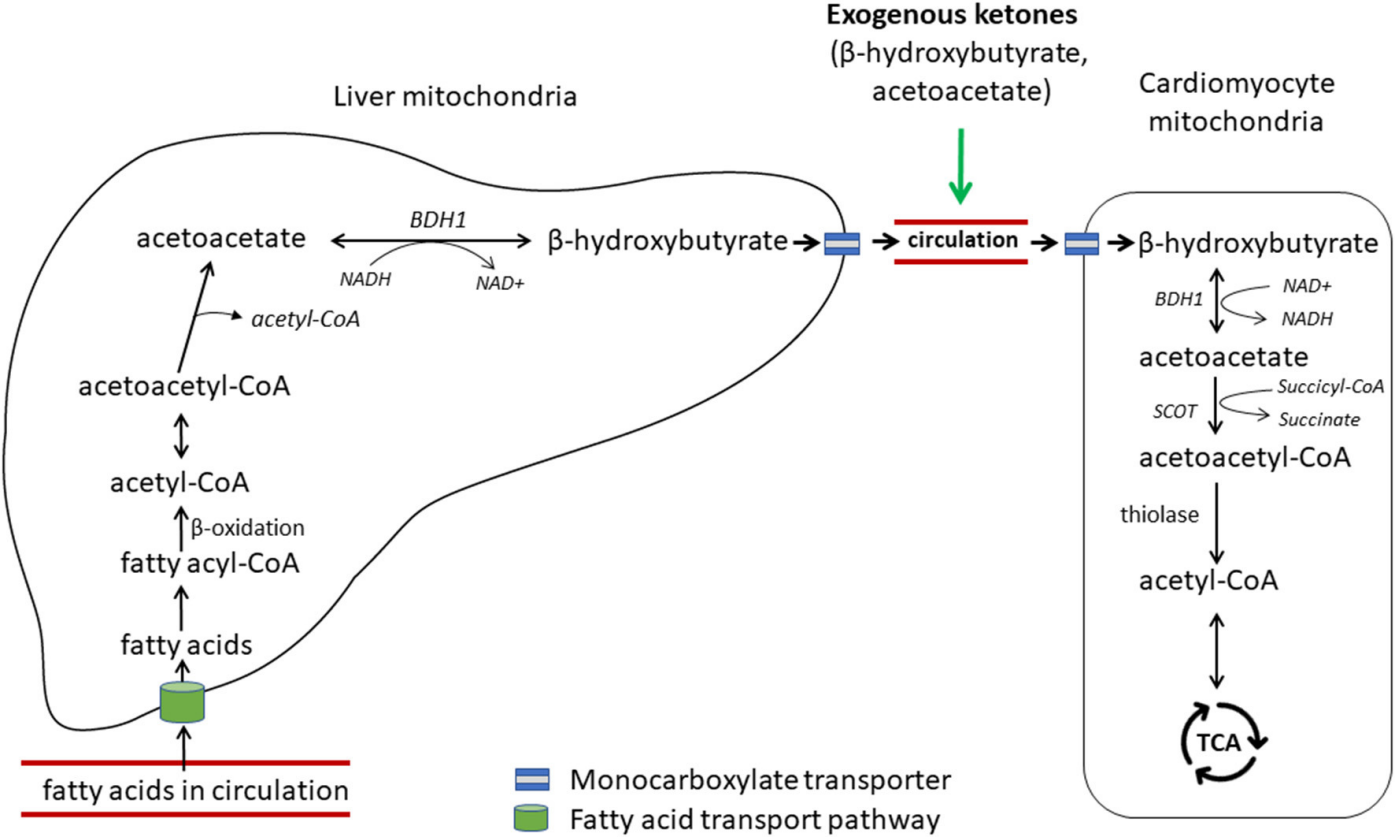
Hepatic ketogenesis and cardiac ketolysis. Free fatty acids in circulation are transported into the liver *via* a carrier protein-assisted transport pathway. These fatty acids undergo fatty acid oxidation in the hepatic mitochondria to produce acetyl-CoA, which after a series of enzymatic reactions produces ketone bodies, acetoacetate and β-hydroxybutyrate. Ketones are transported to the heart and other extrahepatic organs *via* blood portal by the monocarboxylate transporters 1 and 1 (MCT1/2). β-hydroxybutyrate is converted to acetoacetate by β-hydroxybutyrate dehydrogenase 1 (BDH1), one of the key enzymes for ketone utilization. Acetoacetate is activated to acetoacetyl-CoA by succinyl-CoA:3 ketoacid-CoA transferase (SCOT), the rate-limiting enzyme for ketolysis. Acetoacetyl-CoA undergoes a final thiolysis reaction to be separated into 2 molecules of acetyl-CoA. Adapted from Selvaraj et al. (7).

Acetoacetate and β-hydroxybutyrate (BHB) are the two main forms of ketone bodies. Under normal, non-fasting conditions, ketones contribute little to myocardium energy metabolism. Recently, emerging evidence demonstrates the importance of ketones as an alternate fuel source for the failing heart (7, 11, 12, 14). Ketone bodies are mainly produced in the liver cells from circulating fatty acids (Figure 4) (56). After a series of enzymatic reactions, two molecules of acetyl-CoA are converted to one molecule of acetoacetate, which is further reduced to BHB by β-hydroxybutyrate dehydrogenase 1 (BDH1) in the mitochondria. These ketone bodies reach other tissues *via* circulation and are taken up by other organs by monocarboxylate transporters. In cardiomyocytes, BHB is oxidized to be reconverted into acetoacetate by BDH1, a key enzyme for ketone utilization. Acetoacetate is activated by succinyl-CoA:3 ketoacid-CoA transferase (SCOT), the rate-limiting enzyme of ketolysis, to become acetoacetyl-CoA, which undergoes a final round of thiolysis to produce 2 molecules of acetyl-CoA. Acetyl-CoA enters the TCA cycle to fuel energy production.

In humans, blood ketone bodies are elevated in patients with CHF, and are inversely correlated with LV ejection fraction (57, 58). The gene expressions of myocardial BHD1 and SCOT were upregulated in human HF patients compared with non-HF controls (12). In a well-defined mouse model of HF, the expression of BHD1 protein was increased in the hypertrophied and failing heart, and ketone oxidation was increased in the context of reduced FAO (11). More recently, Horton and colleagues demonstrated that the shift to ketone utilization in the failing heart is adaptive, and that BHD1-deficient mice unable to utilize ketones in the heart resulted in worsened HF in response to insults (14). Additionally, mice with increased delivery of ketone bodies by a ketogenic diet or direct ketone infusion to the heart ameliorated pathological cardiac remodeling and dysfunction. These authors further demonstrated ketone bodies as a metabolic stress defense, rendering protective effects on pathologic cardiac remodeling and dysfunction in dogs. Although still in its early development (59), therapeutic ketosis to treat HF starts to gain considerable attention (7, 60).

Similar to human HF patients, the levels of circulating BHB and acetoacetate are also increased in dogs with preclinical MMVD as well as those with CHF compared with healthy dogs (22). Increases in circulating ketone bodies may be the result of a compensatory increase in ketone production in the liver or a decrease in myocardium ketone utilization or both. To date, numerous myocardial and heart valve gene expression studies have been reported in dogs with MMVD (48, 61–63). The gene expression of SCOT was downregulated in the MV in dogs with preclinical MMVD (48). No myocardial expressional change in BDH1 or SCOT gene or protein has been reported. One possibility is that the myocardium and heart valve adapt to different energy substrates in the early phase of MMVD pathogenesis: while the myocardium can metabolize both glucose and ketones for fuel, the heart valve relies primarily on glucose. It would be interesting to test the hypothesis on more MV and myocardial samples.

### OXPHOS in Mitochondria

Mitochondria, the “powerhouse” of the cell, supplies 95% of energy to cardiomyocytes (16, 45). The catabolic products of fatty acids, glucose, ketone bodies, and amino acids are used to fuel the TCA cycle to generate energy substrates, which enter the electron transport chain (ETC) for OXPHOS. Electron transport induces proton pumping from the inner mitochondrial matrix to the mitochondrial intermembrane space, a process that generates the membrane potential for ATP production (64, 65). The levels of circulating citrate and aconitate, both of which are TCA cycle intermediates, are increased in canine MMVD (22). Accumulation of these intermediates in circulation may signify impaired or inefficient TCA cycle. In addition, the concentration of inorganic phosphate (Pi) is elevated in circulation in proportion to the severity of MMVD in dogs (22). Pi is an important regulator of cytosolic ATP production (66, 67). An *in vitro* study demonstrated that Pi plays a complex regulatory role in multiple sites of OXPHOS, including electron flow, generation of reduced forms of nicotinamide adenine dinucleotide (NAD+), distribution of energy flow in the cytochrome chain, as well as serving as the primary substrate for the ATPase to produce ATP in the cardiac mitochondria (68). It is likely that the increased Pi level in MMVD dogs is a part of the cytosolic feedback signaling to preserve energy metabolism homeostasis in the energy-deprived failing heart.

### Creatine Kinase Shuttle and Energy Transfer to Myofibrils

The high-energy phosphate bond in ATP is transferred to creatine by the mitochondrial creatine kinase to generate phosphocreatine (PCr) and ADP. PCr, a molecule smaller than ATP, can rapidly diffuse from mitochondria to myofibrils, where myocardial creatine kinase catalyzes the reconversion from PCr to ATP and release free creatine. Free creatine is recycled in the mitochondria. In mammals, the majority of creatine is obtained from the diet or biosynthesized in the liver and kidneys and is taken up by the heart from circulation against a large concentration gradient using the specific creatine transporter (Figure 1) (69, 70).

In HF, the total myocardial creatine pool size is consistently decreased regardless of species or etiology, possibly due to reduced sarcolemmal creatine uptake (71, 72). Total creatine kinase as well as mitochondrial creatine kinase activity are also reduced in human HF patients and animal models of HF (43). However, the causal relationship between the impaired creatine kinase shuttle pathway and reduced myocardial ATP levels has not yet been established. In dogs with MMVD, the concentrations of circulating creatine are increased as the disease advances (22). Notably, the level of circulating creatine in dogs with stage B1 MMVD is higher than that of healthy dogs. Because the degradation of creatine to creatinine is a slow unregulated process, creatine levels are determined by creatine transporter activity (43). One hypothesis is that increased serum creatine levels are likely the result of reduced sarcolemmal creatine transporter activities, and that myocardial creatine level is decreased at the very early stage of MMVD. This observation is consistent with the hypothesis that cardiac energy deficiency has already begun in the early preclinical stage of canine MMVD. Moderate augmentation of creatine kinase activity to increase creatine and PCr levels in myocardium through pharmaceutical or nutritional intervention has been considered as an attractive strategy (73, 74). However, caution should be taken because massive increases in the creatine transporter function can have detrimental effects (75).

## Amino Acid Metabolisms

### Glutathione

Oxidative stress is an imbalanced state between generation and elimination of reactive oxygen species (ROS). Increased production and decreased removal of ROS play a causal role in the pathophysiology of HF (76). In the heart, mitochondria function as a redox hub (77). Superoxide (${\text{O}}_{2}^{-}$) is generated in the ETC but is quickly converted to oxygen (O_2_) and hydrogen peroxide (H_2_O_2_) by superoxide dismutase. Free H_2_O_2_ is further reduced to water by glutathione peroxidase, consuming two molecules of reduced glutathione (GSH) and generating 1 molecule of oxidized glutathione (GSSG): 2GSH + H_2_O_2_ → GSSG + 2H_2_O. Glutathione peroxidase activity is inversely correlated to the risk of coronary artery disease (78).

Glutathione, a tripeptide of glutamine, cysteine, and glycine, determines intracellular redox state (79). Systemic glutathione relates to HF progression and cardiac remodeling. Myocardial and circulating glutathione levels are depleted in cardiac patients compared with healthy controls (80, 81). In dogs with CHF, the plasma ratio of reduced to oxidized glutathione (GSH:GSSG) is significantly lower than that of healthy controls (82). Circulating GSSG is also higher in dogs with preclinical MMVD compared with healthy dogs (48). For many mammals including humans and dogs, methionine is an essential amino acid that must be supplied through diets, while glycine is a conditionally essential amino acid that cannot not be sufficiently synthesized endogenously and has to be supplemented *via* diets. Methionine serves as the precursor for cysteine, taurine, and carnitine biosynthesis. The concentration of circulating methionine is lower in dogs with preclinical MMVD and CHF compared with healthy dogs (22, 48). Seral concentrations of glycine and glutamine are also reduced in MMVD vs. healthy dogs (22). The key determinants of GSH synthesis are the availability of cysteine and the activity of the rate-limiting enzyme, glutamate cysteine ligase. Decreased methionine, glycine, and glutamine in circulation may signify reduced myocardial GSH biosynthesis in dogs with MMVD.

### Carnitine, Deoxycarnitine, and Acylcarnitines

L-carnitine plays an important role in fatty acid metabolism and oxidation and is concentrated in the skeletal and cardiac muscles. Myocardium can synthesize deoxycarnitine, an immediate precursor of L-carnitine, but lacks the hydroxylase that catalyzes the final conversion from deoxycarnitine to carnitine (83, 84). In mammals, L-carnitine is synthesized from lysine and methionine in the liver, brain, and in human kidneys. There is a bidirectional exchange between carnitine and deoxycarnitine across cardiac sarcolemma: the heart uses its deoxycarnitine to exchange for L-carnitine from the blood stream (83). In human patients with dilated cardiomyopathy (DCM) and CHF, total and free myocardial carnitine levels, and carnitine palmitoyl-transferase (CPT) activities are significantly lower, while plasma total and free carnitine concentrations are higher when compared with healthy controls (85–87). In dogs, myocardial carnitine deficiency was first associated with a family of dogs with DCM (88). Reduced myocardial carnitine and increased plasma carnitine concentration were reported in pacing-induced CHF in adult mongrel dogs (89). Circulating deoxycarnitine is lower in dogs with preclinical MMVD than healthy dogs (48), while total and free carnitine levels are increased in proportion to the severity of MMVD (22, 28). It is possible that the myocardium's ability to synthesize deoxycarnitine is impaired in dogs with MMVD and that its ability to exchange carnitine from the blood stream is compromised, resulting in reduced myocardial carnitine uptake and increased levels of circulating carnitine. Nevertheless, the causal relationship between carnitine deficiency and cardiac disease in dogs has not been established. The benefit of carnitine supplementation in canine heart disease remains observational (90, 91).

Acylcarnitines are intermediates of FAO. Accumulation in acylcarnitines in the blood signifies disorders in mitochondrial or peroxisomal FAO (92, 93). Elevated levels of plasma long-chain (C14–C21), median-chain (C6–C13), and short-chain (C2–C5) acylcarnitines were documented in human HF patients (94–96). Accumulation of long-chain acylcarnitines in circulation is thought to contribute to the pathogenesis of HF by stimulating ROS production and releasing inflammatory mediators (95). Chen et al. showed that human patients with acute HF had higher plasma levels of acylcarnitines of all types, compared with normal controls (96). Improved FAO was associated with improved cardiac function along with substantial decreases in plasma long-chain and short-chain acylcarnitines (96). In dogs with MMVD, twenty-two long-chain, medium-chain, and short-chain acylcarnitines are accumulated in circulation in MMVD dogs vs. healthy dogs (22). Short-chain acylcarnitines are the degradation products of BCAAs, derived from muscular breakdown or gut microbiota metabolism. Accumulation of adipoylcarnitine (C6-DC), a dicarboxylcarnitine and several hydroxyl-acylcarnitines suggests activation of ω-FAO in peroxisome, which is a rescue pathway in response to impaired mitochondrial β-oxidation (97). Carnitine and acylcarnitines are positively correlated with one another, and with left atrial dimension in dogs (22). Remarkably, in a 6-month diet intervention study where improvements in left atrial enlargement and mitral regurgitation were observed in dogs with preclinical MMVD (18), three circulating acylcarnitines, oleoylcarnitine (C18), adipoylcarnitine (C6-DC), and margaroylcarnitine (C17), were decreased in dogs fed the intervention diet, while little change was observed in dogs fed the control diet (98). In the same study, the seral level of deoxycarnitine was increased in response to the diet intervention (98). The utility of free carnitine or carnitine esters as diagnostic or prognostic biomarkers for canine MMVD warrants further investigation.

### Tryptophan Metabolism

**Figure 5:**
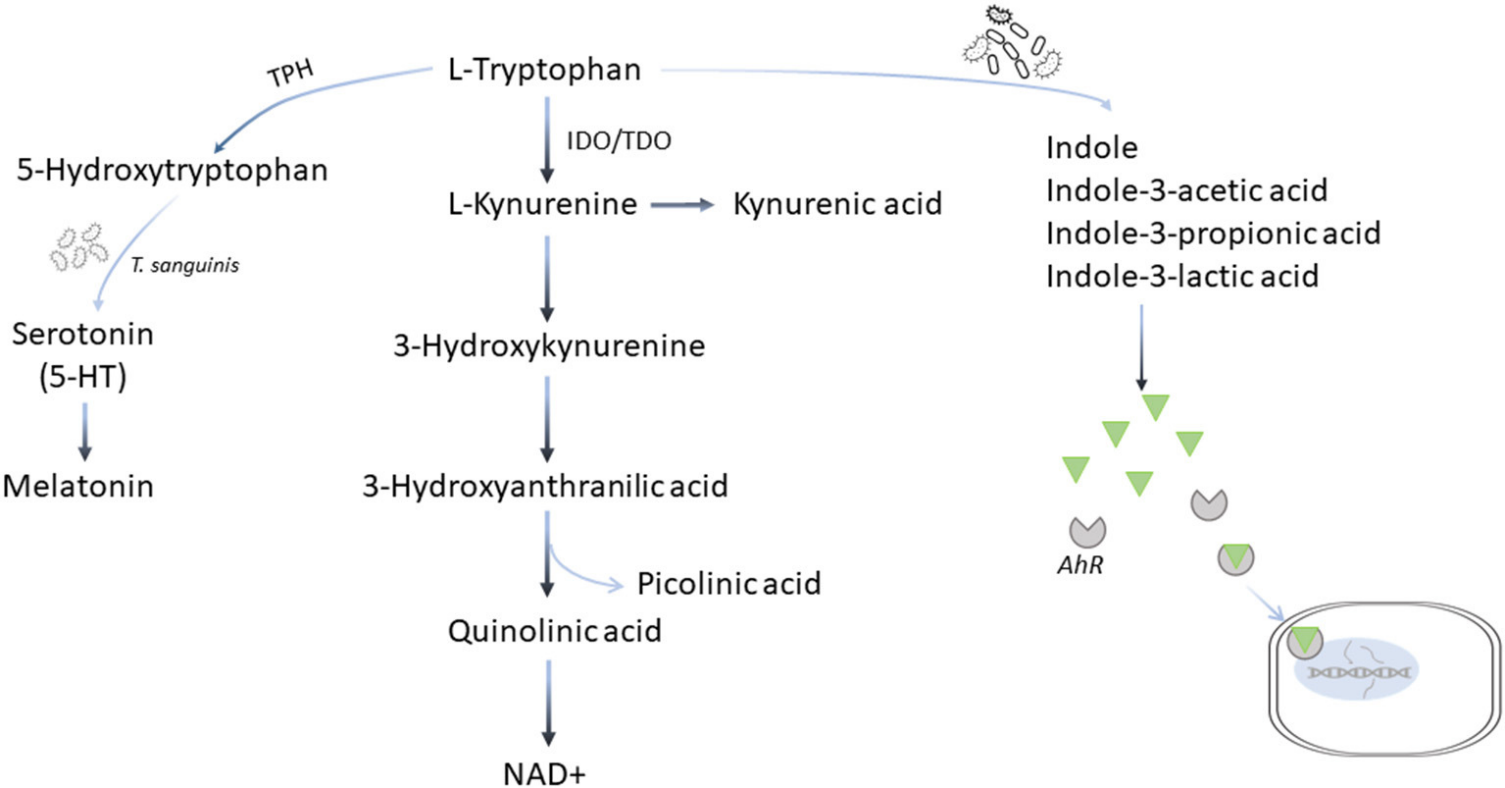
Tryptophan metabolic pathways. Tryptophan is metabolized by three pathways. More than 90% of dietary tryptophan is metabolized *via* the main kynurenine pathway mediated by the indoleamine 2,3-dioxygenase (IDO) and tryptophan 2,3-deoxygenase (TDO) leading to the production of important metabolites, such as kynurenine, kynurenic acid, quinolinic acid, and eventually nicotinamide adenine dinucleotide (NAD+), and picolinic acid; the minor serotonin (5-HT) pathway *via* tryptophan hydroxylase (TPH), and the third the microbiota-dependent pathways to produce several key metabolites including ligands for the aryl hydrocarbon receptor (AhR)-mediated signaling, indole and its derivative.

Tryptophan (Trp) is another essential amino acid that must be acquired through diet in both humans and dogs (99, 100). In addition to protein synthesis, dietary Trp is metabolized by three pathways. The main kynurenine pathway *via* indoleamine 2,3-dioxygenase (IDO) and tryptophan 2,3-dioxygenase (TDO) leads to the production of important metabolites, such as kynurenine (Kyn), kynurenic acid (KA), quinolinic acid (QA), and eventually nicotinamide adenine dinucleotide (NAD+), and picolinic acid; the minor serotonin (5-HT) pathway *via* Trp hydroxylase (TPH), and the third the microbiota-dependent pathways to produce several key metabolites including ligands for the aryl hydrocarbon receptor (AhR)-mediated signaling, indole and its derivatives (Figure 5) (101–103). Human patients with cardiovascular disease and CHF often have accelerated Trp catabolism leading to lower circulating Trp levels and higher Kyn/Trp ratios compared with healthy individuals (104–108). In dogs with MMVD, although no change in Trp or Kyn is observed, the concentrations of QA are increased in MMVD dogs compared with healthy dogs, suggesting an upregulation in the Trp-Kyn pathway (22).

Upregulation of the Trp-Kyn pathway may also lead to increased production of NAD+, an essential cofactor for bioenergetics and an important coenzyme for FAO, glycolysis, TCA cycle, and ETC. In mammalian cells, the majority of NAD+ is produced by the salvage pathway that recycles nicotinamide and nicotinamide riboside to NAD+, while the remaining balance comes from *de novo* biosynthesis *via* the Trp-Kyn pathway, and the Preiss-Handler pathway using nicotinic acid (109). Seral concentration of nicotinamide, precursor for the salvage pathway, is decreased, while that of QA, a key intermediate of the *de novo* pathway, is increased in dogs with MMVD compared with healthy dogs (22). These results suggest that the main salvage pathway for NAD+ production may be compromised, while the *de novo* biosynthesis *via* the Trp-Kyn pathway is activated to rescue (22). However, QA and 3-hydroxykynurenine, both of which are Kyn metabolites with cytotoxicity, may directly interfere with mitochondrial function by AhR activation, and intensify the ROS production leading to mitochondrial impairment (110–112).

Trp is a substrate for TPH, a rate-limiting enzyme that hydroxylates Trp to form 5-HT, which enters the cells through serotonin transporter (SERT) (113). Serotonin has been associated with pathological remodeling in mature human heart valves (114–116). In particular, serotonergic 5-HT_2_ receptors are implicated in heart disease (117, 118). The 5-HT signaling pathway has been linked to the pathogenesis of MMVD in dogs (39). Circulating 5-HT is increased in early stage MMVD but decreased as the disease progresses to end stage (38, 41). However, in the untargeted serum metabolomics studies comparing healthy dogs and dogs with different stages of MMVD, no difference in 5-HT was observed (22, 48). More than 95% of 5-HT in the body is produced in the gut. *Turicibacter sanguinis*, a spore-forming bacteria in the gut, signals intestinal enterochromaffin cells to produce 5-HT (102, 119). In a recent fecal microbiome study using the 16S rRNA gene sequencing, at the genus level the abundances of *Turicibacter* are reduced in dogs with MMVD compared with healthy dogs (120). However, the sequencing method did not provide enough resolution to identify the species of *Turicibacter*. The nature of the association requires further investigation.

### BCAA in Heart Failure

Energy substrate readaptation is one of the hallmarks of the failing heart. While much attention has been focused on the regulatory mechanism and functional impacts of fatty acids and carbohydrates, the contribution of amino acid metabolism in the development of HF is largely understudied (25). In an early study, Peterson et al. demonstrated that myocardial free amino acids were increased in human HF (23). Several amino acids including BCAAs, were increased in circulation in a rat model of hypertension (121). Metabolomics and transcriptomics studies also revealed changes in BCAAs and key amino acid metabolic pathways in murine models of HF (21, 122). Sun and colleagues reported that catabolic deficiency of BCAA, including leucine, isoleucine, and valine, is a metabolic hallmark for murine failing heart and human DCM (24). It was postulated that BCAA catabolic deficiency leads to accumulation of branched-chain alpha-keto acids, induces ROS, and activates mTOR (25). In dogs, a recent untargeted serum metabolomics study reported accumulations of numerous intermediates of BCAA metabolism in MMVD (22). The quantitative Metabolite Set Enrichment Analysis indicated enrichment of oxidation of BCAAs in MMVD dogs with CHF vs. healthy dogs (22). Circulating valine concentration was slightly lower in preclinical dogs with MMVD compared to healthy controls (48). The nature of the association between BCAA metabolism and canine heart disease, if any, warrants further investigation.

### Taurine

Taurine is one of the sulfur-containing amino acids that is not incorporated into proteins but found to be in high concentrations in the heart and skeletal muscles (123). In mammals, the susceptibility to taurine deficiency varies by species: while taurine can be synthesized endogenously and is considered non-essential or conditionally-essential in humans, rodents, and dogs, it is essential for cats (91, 124–127). Taurine has been implicated in the maintenance of normal contractile function, modulation of myocardial calcium homeostasis, and potentially acts as an antioxidant and anti-inflammatory agent (123, 128). Schaffer et al. demonstrated that the taurine-deficient heart is associated with reduced ATP generation and is energy starved, possibly due to impaired mitochondrial respiratory chain activity, and NADH utilization (129). Although taurine deficiency causes reversible cardiomyopathy in cats (130), it does not play a significant role in the development of cardiomyopathy in dogs (91). Freeman et al. found no correlation between dietary and circulating taurine concentrations (131). A retrospective study on DCM in dogs suggested that taurine supplementation was not associated with survival or echocardiographic changes although the study did not rule out the possibility of a breed-specific role of taurine (132). In untargeted serum metabolomics studies, no difference in taurine concentration was found between healthy dogs and dogs with various stages of MMVD (22, 48).

## GUT Dysbiosis and MMVD

The human gastrointestinal tract is colonized with 10–100 trillions of typically non-pathogenic commensal microorganisms, collectively known as microbiota (133). These microorganisms encode >4 million non-redundant genes, which is more than 100 times of human genomes (134, 135). The additional pool of microbial genes aid in food digestion and absorption, xenobiotic metabolism, development of immune system (136), and contribute to the pathogenesis of metabolic disorders, including cardiovascular disease (29, 30). Since the establishment of an initial link between gut microbiota and cardiovascular disease (CVD), numerous gut microbiota-dependent metabolites and pathways, including the trimethylamine N-oxide (TMAO) pathway, short-chain fatty acid pathways, and bile acid pathways, have been implicated in the pathogenesis of CVD and HF (29, 30, 137–139). The gut hypothesis of HF postulates that impaired intestinal mucosal integrity in HF patients allows gut bacteria and their endotoxins to leak into circulation, and resultant chronic and low-grade systemic inflammation characteristic of HF (31, 138, 139). TMAO, a diet-derived metabolite that is coproduced by microbiota and host, has been associated with cardiovascular diseases including HF (29, 30, 139). Dietary precursors, including L-carnitine, choline/phosphatidylcholine, and to a less degree, betaine, are converted to trimethylamine (TMA) by gut microbiota. TMA enters the portal circulation to be further oxidized to form TMAO by host hepatic enzymes known as flavin-containing monooxygenases (FMOs) (30, 139). Trimethyllysine, a methylated derived of amino acid lysine, is another source for the endogenous TMAO synthesis although less efficient than TMA (140). In dogs with preclinical MMVD and CHF, circulating TMAO as well as its nutritional precursors, L-carnitine, phosphatidylcholines and betaines, are increased compared with healthy controls (22, 28). A recent study analyzing fecal microbiome in healthy dogs and dogs with MMVD shows that gut microbial diversities are significantly different between healthy dogs and dogs with CHF (120). The dysbiosis index, which is measured using quantitative PCR on a panel of eight fecal bacterial groups, shows increases at the pre-clinical stages and becomes significantly higher in dogs with CHF when compared with healthy dogs. The study suggests that gut microbiota change has begun at the early preclinical MMVD. Significant differences in the abundance of *E. coli*, were found between dogs with MMVD vs. healthy dogs (120, 141). The *E. coli* genome shares 99% sequence identity with carnitine oxygenase (*cntA*), the key gene for TMA biosynthesis (142). It is possible that *E. coli* contributes to the increase in TMAO in MMVD dogs. However, the causal link between cardiovascular diseases and TMAO or its dietary precursors has yet to be determined. The abundance of *C. hiranonis*, a gut bacterium capable of converting primary bile acid to secondary bile acid, is inversely associated with dysbiosis index. Strikingly, the bile acid conversion was complete in dogs with high levels *C. hiranonis*, but incomplete in those without (120). The preliminary data indicate an interplay among host, gut microbiota, and signaling pathways mediated by the gut microbe-dependent metabolites in MMVD in dogs.

## Nutrition Intervention to Address Metabolic Changes

Nutrition plays an important role in heart health (143). Sodium restriction has been recommended to human patients with HF due to its ability to lower blood pressure and prevent hypertension (144). However, multiple randomized controlled studies in humans demonstrated that sodium restrictions activate renin-angiotensin-aldosterone system (RAAS) and increase insulin resistance. The existing evidence does not support a universal reduction in sodium intake in CVD patients (145, 146). Roles of caloric restriction, omega-3 PUFAs, taurine, carnitine, B vitamins, magnesium, potassium, coenzyme Q10, and antioxidants in human and canine CVD have also been extensively discussed (90, 131, 143, 147–150), but most of the benefits remain observational or from case reports. Rigorous randomized controlled studies are warranted. The use of low-sodium diets in dogs with HF is a common practice for veterinarians, but the advantages and disadvantages of sodium restriction on canine patients with CHF warrants further investigations (151). One concern is that the RAAS signaling has vasoconstrictor properties and is thought to contribute to renal injury. In one study, low salt diet induces RAAS, increases oxidative stress and attenuates nitric oxide bioavailability in the canine heart (152). In a double-blinded, crossover study, 18 dogs with HF were randomized into either a low-sodium diet or a moderate-sodium diet for 4 weeks. Among the dogs that completed the study, maximal left ventricular size showed a marginal decrease on the low-sodium diet (*P* = 0.05) (151). The same research group followed up with a 4-week randomized placebo-controlled study to test the efficacy of a moderately reduced sodium diet enriched with omega-3 PUFAs, carnitine, taurine, arginine, and several antioxidants in 29 dogs with asymptomatic preclinical MMVD (153). The report didn't state whether the study was blinded or not. Dogs fed the test diet had significant reductions in maximal left atrial diameter (both weight-based and non-weight-based) and left ventricular internal dimension in diastole (non-weight-based) compared with the placebo controls (153). In recent years, the systems biology approach has been increasingly used to probe the molecular and metabolic pathways underlying cardiovascular diseases and to generate testable hypotheses to address those changes (48, 154). A cardiac protection blend of nutrients (CPB), including medium-chain triglycerides, fish oil, amino acids taurine, methionine, lysine, magnesium, and vitamin E, was designed based on the results of a multi-omics study on canine MMVD (18, 48). In a 6-month, single-blinded, randomized, placebo-controlled dietary intervention study, Li et al. tested the clinical efficacy of CPB on preclinical dogs with MMVD (18). Dogs supplemented with CPB had significant reductions in left atrial diameter, left atrial to aortic root ratio, and the severity of mitral regurgitation when compared with dogs fed the placebo diet. Notably, several dogs in the placebo group advanced from B1 stage to B2 stage at 6 months, while no dog in the CPB group progressed from B1 to B2 (*P* < 0.05). Untargeted metabolomics study using the serum samples from these dogs supported the hypothesis that CPB improves energy metabolism and reduces inflammation and oxidative stress (98). Large studies with more dogs from different breeds should be conducted to confirm the results. Several micronutrients are essential for mitochondrial health, energy metabolism and production. In the TCA cycle, vitamin B1 (thiamine) is part of pyruvate dehydrogenase complex for the conversion from pyruvate to acetyl-CoA, vitamin B5 (pantothenic acid) is a precursor for coenzyme A biosynthesis, and vitamin B12 (cobalamin) is a cofactor for succinyl-CoA formation. Some nutrients are also crucial for the activities of the ETC complex: vitamin B3 (niacin) is a precursor of NAD+ biosynthesis, vitamin B2 (riboflavin) is a building block for ETC complex I and II, and coenzyme Q10 and taurine are associated with the activities of ETC complex. Amino acid metabolic readaptation in the failing heart provides additional opportunities for nutrition intervention. The level of methionine, an essential amino acid, is lower in dogs with MMVD than control dogs. The catabolism of glucogenic and ketogenic amino acids can generate glucose, ketones and other energy substrates important for energy homeostasis. Finally, supplementation of certain prebiotic fibers can be used to reduce uremic toxins including TMAO and restore gut symbiosis, and provides an alternative therapeutic option for canine heart patients.

Many nutrition intervention studies were designed to test combinations of nutrients, which can perform better than individual supplements (155, 156). *In vitro or in vivo* models may be used to understand the roles each nutrient plays or how they interact. These models can also be used to screen for nutrients or combination of nutrients for synergistic effects before clinical testing.

## Concluding Remarks

Recent advances in systems biology and high-throughput multi-omics technologies make it possible to explore molecular and metabolic changes at the systems level in canine MMVD and HF. Some of the cellular and metabolic pathways are excellent targets for nutritional or pharmaceutical interventions. As our knowledge in systems biology and nutrition science continues to grow and with the new technologies and diagnostics available, there will exist significant opportunities to deliver breakthrough nutritional interventions to support dogs with MMVD and other cardiac diseases.

## Author Contributions

QL is responsible for the conception and writing of the manuscript.

## Conflict of Interest

QL is a current employee of Nestlé Purina PetCare Company.

## Publisher's Note

All claims expressed in this article are solely those of the authors and do not necessarily represent those of their affiliated organizations, or those of the publisher, the editors and the reviewers. Any product that may be evaluated in this article, or claim that may be made by its manufacturer, is not guaranteed or endorsed by the publisher.

## References

[1] Lopaschuk GD Ussher JR Folmes CD Jaswal JS Stanley WC. Myocardial fatty acid metabolism in health and disease. Physiol Rev. (2010) 90:207–58. 10.1152/physrev.00015.2009 20086077

[2] Dorn GW II Vega RB Kelly DP. Mitochondrial biogenesis and dynamics in the developing and diseased heart. Genes Dev. (2015) 29:1981–91. 10.1101/gad.269894.115 26443844 PMC4604339

[3] Herrmann G Decherd G. The chemical nature of heart failure. Ann Intern Med. (1939) 12:1233–44. 10.7326/0003-4819-12-8-1233

[4] Lopaschuk GD Belke DD Gamble J Itoi T Schonekess BO. Regulation of fatty acid oxidation in the mammalian heart in health and disease. Biochim Biophys Acta. (1994) 1213:263–76. 10.1016/0005-2760(94)00082-4 8049240

[5] Stanley WC Recchia FA Lopaschuk GD. Myocardial substrate metabolism in the normal and failing heart. Physiol Rev. (2005) 85:1093–129. 10.1152/physrev.00006.2004 15987803

[6] Neubauer S. The failing heart–an engine out of fuel. N Engl J Med. (2007) 356:1140–51. 10.1056/NEJMra063052 17360992

[7] Selvaraj S Kelly DP Margulies KB. Implications of altered ketone metabolism and therapeutic ketosis in heart failure. Circulation. (2020) 141:1800–12. 10.1161/CIRCULATIONAHA.119.045033 32479196 PMC7304522

[8] Neubauer S Horn M Cramer M Harre K Newell JB Peters W. Myocardial phosphocreatine-to-ATP ratio is a predictor of mortality in patients with dilated cardiomyopathy. Circulation. (1997) 96:2190–6. 10.1161/01.CIR.96.7.2190 9337189

[9] Lionetti V Stanley WC Recchia FA. Modulating fatty acid oxidation in heart failure. Cardiovasc Res. (2011) 90:202–9. 10.1093/cvr/cvr038 21289012 PMC3078800

[10] Tran DH Wang ZV. Glucose metabolism in cardiac hypertrophy and heart failure. J Am Heart Assoc. (2019) 8:e012673. 10.1161/JAHA.119.012673 31185774 PMC6645632

[11] Aubert G Martin OJ Horton JL Lai L Vega RB Leone TC. The failing heart relies on ketone bodies as a fuel. Circulation. (2016) 133:698–705. 10.1161/CIRCULATIONAHA.115.017355 26819376 PMC4766035

[12] Bedi KC Jr Snyder NW Brandimarto J Aziz M Mesaros C Worth AJ. Evidence for intramyocardial disruption of lipid metabolism and increased myocardial ketone utilization in advanced human heart failure. Circulation. (2016) 133:706–16. 10.1161/CIRCULATIONAHA.115.017545 26819374 PMC4779339

[13] Uchihashi M Hoshino A Okawa Y Ariyoshi M Kaimoto S Tateishi S. Cardiac-Specific Bdh1 overexpression ameliorates oxidative stress and cardiac remodeling in pressure overload-induced heart failure. Circ Heart Fail. (2017) 10:e007405. 10.1161/CIRCHEARTFAILURE.117.004417 29242353

[14] Horton JL Davidson MT Kurishima C Vega RB Powers JC Matsuura TR. The failing heart utilizes 3-hydroxybutyrate as a metabolic stress defense. JCI Insight. (2019) 4:e124079. 10.1172/jci.insight.124079 30668551 PMC6478419

[15] Mann DL Bristow MR. Mechanisms and models in heart failure: the biomechanical model and beyond. Circulation. (2005) 111:2837–49. 10.1161/CIRCULATIONAHA.104.500546 15927992

[16] Brown DA Perry JB Allen ME Sabbah HN Stauffer BL Shaikh SR. Expert consensus document: mitochondrial function as a therapeutic target in heart failure. Nat Rev Cardiol. (2017) 14:238–50. 10.1038/nrcardio.2016.203 28004807 PMC5350035

[17] Saifudeen I Subhadra L Konnottil R Nair RR. Metabolic modulation by medium-chain triglycerides reduces oxidative stress and ameliorates CD36-mediated cardiac remodeling in spontaneously hypertensive rat in the initial and established stages of hypertrophy. J Card Fail. (2017) 23:240–51. 10.1016/j.cardfail.2016.08.001 27530817

[18] Li Q Heaney A Langenfeld-Mccoy N Boler BV Laflamme DP. Dietary intervention reduces left atrial enlargement in dogs with early preclinical myxomatous mitral valve disease: a blinded randomized controlled study in 36 dogs. BMC Vet Res. (2019) 15:425. 10.1186/s12917-019-2169-1 31775756 PMC6882217

[19] Nielsen R Moller N Gormsen LC Tolbod LP Hansson NH Sorensen J. Cardiovascular effects of treatment with the ketone body 3-hydroxybutyrate in chronic heart failure patients. Circulation. (2019) 139:2129–41. 10.1161/CIRCULATIONAHA.118.036459 30884964 PMC6493702

[20] Venturini A Ascione R Lin H Polesel E Angelini GD Suleiman MS. The importance of myocardial amino acids during ischemia and reperfusion in dilated left ventricle of patients with degenerative mitral valve disease. Mol Cell Biochem. (2009) 330:63–70. 10.1007/s11010-009-0101-x 19363596 PMC2850556

[21] Lai L Leone TC Keller MP Martin OJ Broman AT Nigro J. Energy metabolic reprogramming in the hypertrophied and early stage failing heart: a multisystems approach. Circ Heart Fail. (2014) 7:1022–31. 10.1161/CIRCHEARTFAILURE.114.001469 25236884 PMC4241130

[22] Li Q Larouche-Lebel E Loughran KA Huh TP Suchodolski JS Oyama MA. Metabolomics analysis reveals deranged energy metabolism and amino acid metabolic reprogramming in dogs with myxomatous mitral valve disease. J Am Heart Assoc. (2021) 10:e018923. 10.1161/JAHA.120.018923 33890477 PMC8200728

[23] Peterson MB Mead RJ Welty JD. Free amino acids in congestive heart failure. J Mol Cell Cardiol. (1973) 5:139–47. 10.1016/0022-2828(73)90047-3 4704669

[24] Sun H Olson KC Gao C Prosdocimo DA Zhou M Wang Z. Catabolic defect of branched-chain amino acids promotes heart failure. Circulation. (2016) 133:2038–49. 10.1161/CIRCULATIONAHA.115.020226 27059949 PMC4879058

[25] Sun H Wang Y. Branched chain amino acid metabolic reprogramming in heart failure. Biochim Biophys Acta. (2016) 1862:2270–5. 10.1016/j.bbadis.2016.09.009 27639835

[26] Wang W Zhang F Xia Y Zhao S Yan W Wang H. Defective branched chain amino acid catabolism contributes to cardiac dysfunction and remodeling following myocardial infarction. Am J Physiol Heart Circ Physiol. (2016) 311:H1160–9. 10.1152/ajpheart.00114.2016 27542406

[27] Uddin GM Zhang L Shah S Fukushima A Wagg CS Gopal K. Impaired branched chain amino acid oxidation contributes to cardiac insulin resistance in heart failure. Cardiovasc Diabetol. (2019) 18:86. 10.1186/s12933-019-0892-3 31277657 PMC6610921

[28] Karlin ET Rush JE Freeman LM. A pilot study investigating circulating trimethylamine N-oxide and its precursors in dogs with degenerative mitral valve disease with or without congestive heart failure. J Vet Intern Med. (2019) 33:46–53. 10.1111/jvim.15347 30511765 PMC6335534

[29] Wang Z Klipfell E Bennett BJ Koeth R Levison BS Dugar B. Gut flora metabolism of phosphatidylcholine promotes cardiovascular disease. Nature. (2011) 472:57–63. 10.1038/nature09922 21475195 PMC3086762

[30] Tang WH Kitai T Hazen SL. Gut microbiota in cardiovascular health and disease. Circ Res. (2017) 120:1183–96. 10.1161/CIRCRESAHA.117.309715 28360349 PMC5390330

[31] Mamic P Chaikijurajai T Tang WHW. Gut microbiome - a potential mediator of pathogenesis in heart failure and its comorbidities: state-of-the-art review. J Mol Cell Cardiol. (2021) 152:105–17. 10.1016/j.yjmcc.2020.12.001 33307092 PMC7981261

[32] Atkins C Bonagura J Ettinger S Fox P Gordon S Haggstrom J. Guidelines for the diagnosis and treatment of canine chronic valvular heart disease. J Vet Intern Med. (2009) 23:1142–50. 10.1111/j.1939-1676.2009.0392.x 19780929

[33] Keene BW Atkins CE Bonagura JD Fox PR Haggstrom J Fuentes VL. ACVIM consensus guidelines for the diagnosis and treatment of myxomatous mitral valve disease in dogs. J Vet Intern Med. (2019) 33:1127–40. 10.1111/jvim.15488 30974015 PMC6524084

[34] Pomerance A Whitney JC. Heart valve changes common to man and dog: a comparative study. Cardiovasc Res. (1970) 4:61–6. 10.1093/cvr/4.1.61 5416844

[35] Kogure K. Pathology of chronic mitral valvular disease in the dog. Nihon Juigaku Zasshi. (1980) 42:323–35. 10.1292/jvms1939.42.323 7218618

[36] Pedersen HD Haggstrom J. Mitral valve prolapse in the dog: a model of mitral valve prolapse in man. Cardiovasc Res. (2000) 47:234–43. 10.1016/S0008-6363(00)00113-9 10946060

[37] Oyama MA Elliott C Loughran KA Kossar AP Castillero E Levy RJ. Comparative pathology of human and canine myxomatous mitral valve degeneration: 5HT and TGF-beta mechanisms. Cardiovasc Pathol. (2020) 46:107196. 10.1016/j.carpath.2019.107196 32006823 PMC7078050

[38] Arndt JW Reynolds CA Singletary GE Connolly JM Levy RJ Oyama MA. Serum serotonin concentrations in dogs with degenerative mitral valve disease. J Vet Intern Med. (2009) 23:1208–13. 10.1111/j.1939-1676.2009.0378.x 19709352

[39] Oyama MA Levy RJ. Insights into serotonin signaling mechanisms associated with canine degenerative mitral valve disease. J Vet Intern Med. (2010) 24:27–36. 10.1111/j.1939-1676.2009.0411.x 19912520

[40] Orton EC Lacerda CM Maclea HB. Signaling pathways in mitral valve degeneration. J. Vet. Cardiol. (2012) 14:7–17. 10.1016/j.jvc.2011.12.001 22364692

[41] Ljungvall I Hoglund K Lilliehook I Oyama MA Tidholm A Tvedten H. Serum serotonin concentration is associated with severity of myxomatous mitral valve disease in dogs. J Vet Intern Med. (2013) 27:1105–12. 10.1111/jvim.12137 23865457

[42] Cremer SE Singletary GE Olsen LH Wallace K Haggstrom J Ljungvall I. Serotonin concentrations in platelets, plasma, mitral valve leaflet, and left ventricular myocardial tissue in dogs with myxomatous mitral valve disease. J Vet Intern Med. (2014) 28:1534–40. 10.1111/jvim.12420 25146933 PMC4895588

[43] Peterzan MA Lygate CA Neubauer S Rider OJ. Metabolic remodeling in hypertrophied and failing myocardium: a review. Am J Physiol Heart Circ Physiol. (2017) 313:H597–616. 10.1152/ajpheart.00731.2016 28646030

[44] Bertero E Maack C. Metabolic remodelling in heart failure. Nat Rev Cardiol. (2018) 15:457–70. 10.1038/s41569-018-0044-6 29915254

[45] De Jong KA Lopaschuk GD. Complex energy metabolic changes in heart failure with preserved ejection fraction and heart failure with reduced ejection fraction. Can J Cardiol. (2017) 33:860–71. 10.1016/j.cjca.2017.03.009 28579160

[46] Degens H De Brouwer KF Gilde AJ Lindhout M Willemsen PH Janssen BJ. Cardiac fatty acid metabolism is preserved in the compensated hypertrophic rat heart. Basic Res Cardiol. (2006) 101:17–26. 10.1007/s00395-005-0549-0 16136293

[47] Ingwall JS. Energy metabolism in heart failure and remodelling. Cardiovasc Res. (2009) 81:412–9. 10.1093/cvr/cvn301 18987051 PMC2639129

[48] Li Q Freeman LM Rush JE Huggins GS Kennedy AD Labuda JA. Veterinary medicine and multi-omics research for future nutrition targets: metabolomics and transcriptomics of the common degenerative mitral valve disease in dogs. OMICS. (2015) 19:461–70. 10.1089/omi.2015.0057 26154239

[49] Randle PJ Garland PB Hales CN Newsholme EA. The glucose fatty-acid cycle. *Its role in insulin sensitivity and the metabolic disturbances of diabetes mellitus*. Lancet. (1963) 1:785–9. 10.1016/S0140-6736(63)91500-9 13990765

[50] Zhang L Jaswal JS Ussher JR Sankaralingam S Wagg C Zaugg M. Cardiac insulin-resistance and decreased mitochondrial energy production precede the development of systolic heart failure after pressure-overload hypertrophy. Circ Heart Fail. (2013) 6:1039–48. 10.1161/CIRCHEARTFAILURE.112.000228 23861485

[51] Thorens B Mueckler M. Glucose transporters in the 21st century. Am J Physiol Endocrinol Metab. (2010) 298:E141–5. 10.1152/ajpendo.00712.2009 20009031 PMC2822486

[52] De Vos A Heimberg H Quartier E Huypens P Bouwens L Pipeleers D. Human and rat beta cells differ in glucose transporter but not in glucokinase gene expression. J Clin Invest. (1995) 96:2489–95. 10.1172/JCI118308 7593639 PMC185903

[53] Grover-Mckay M Walsh SA Thompson SA. Glucose transporter 3 (GLUT3) protein is present in human myocardium. Biochim Biophys Acta. (1999) 1416:145–54. 10.1016/S0005-2736(98)00216-8 9889355

[54] Simpson IA Dwyer D Malide D Moley KH Travis A Vannucci SJ. The facilitative glucose transporter GLUT3: 20 years of distinction. Am J Physiol Endocrinol Metab. (2008) 295:E242–53. 10.1152/ajpendo.90388.2008 18577699 PMC2519757

[55] Byrne FL Olzomer EM Brink R Hoehn KL. Knockout of glucose transporter GLUT6 has minimal effects on whole body metabolic physiology in mice. Am J Physiol Endocrinol Metab. (2018) 315:E286–93. 10.1152/ajpendo.00082.2018 29664675

[56] Puchalska P Crawford PA. Multi-dimensional roles of ketone bodies in fuel metabolism, signaling, and therapeutics. Cell Metab. (2017) 25:262–84. 10.1016/j.cmet.2016.12.022 28178565 PMC5313038

[57] Lommi J Kupari M Koskinen P Naveri H Leinonen H Pulkki K. Blood ketone bodies in congestive heart failure. J Am Coll Cardiol. (1996) 28:665–72. 10.1016/0735-1097(96)00214-8 8772754

[58] Lommi J Koskinen P Naveri H Harkonen M Kupari M. Heart failure ketosis. J Intern Med. (1997) 242:231–8. 10.1046/j.1365-2796.1997.00187.x 9350168

[59] Ho KL Karwi QG Wagg C Zhang L Vo K Altamimi T. Ketones can become the major fuel source for the heart but do not increase cardiac efficiency. Cardiovasc Res. (2021) 117:1178–87. 10.1093/cvr/cvaa143 32402081 PMC7982999

[60] Lopaschuk GD Karwi QG Ho KL Pherwani S Ketema EB. Ketone metabolism in the failing heart. Biochim Biophys Acta Mol Cell Biol Lipids. (2020) 1865:158813. 10.1016/j.bbalip.2020.158813 32920139

[61] Oyama MA Chittur SV. Genomic expression patterns of mitral valve tissues from dogs with degenerative mitral valve disease. Am J Vet Res. (2006) 67:1307–18. 10.2460/ajvr.67.8.1307 16881841

[62] Lu CC Liu MM Culshaw G Clinton M Argyle DJ Corcoran BM. Gene network and canonical pathway analysis in canine myxomatous mitral valve disease: a microarray study. Vet J. (2015) 204:23–31. 10.1016/j.tvjl.2015.02.021 25841900

[63] Markby GR Summers KM Macrae VE Corcoran BM. Comparative Transcriptomic profiling and gene expression for myxomatous mitral valve disease in the dog and human. Vet Sci. (2017) 4:34. 10.3390/vetsci4030034 29056693 PMC5644653

[64] Zhao RZ Jiang S Zhang L Yu ZB. Mitochondrial electron transport chain, ROS generation and uncoupling (review). Int J Mol Med. (2019) 44:3–15. 10.3892/ijmm.2019.4188 31115493 PMC6559295

[65] Nolfi-Donegan D Braganza A Shiva S. Mitochondrial electron transport chain: oxidative phosphorylation, oxidant production, and methods of measurement. Redox Biol. (2020) 37:101674. 10.1016/j.redox.2020.101674 32811789 PMC7767752

[66] Lardy HA Wellman H. Oxidative phosphorylations; role of inorganic phosphate and acceptor systems in control of metabolic rates. J Biol Chem. (1952) 195:215–24. 10.1016/S0021-9258(19)50892-4 14938372

[67] Chance B Williams GR. The respiratory chain and oxidative phosphorylation. Adv Enzymol Relat Subj Biochem. (1956) 17:65–134. 10.1002/9780470122624.ch2 13313307

[68] Bose S French S Evans FJ Joubert F Balaban RS. Metabolic network control of oxidative phosphorylation: multiple roles of inorganic phosphate. J Biol Chem. (2003) 278:39155–65. 10.1074/jbc.M306409200 12871940

[69] Guimbal C Kilimann MW. A Na(+)-dependent creatine transporter in rabbit brain, muscle, heart, and kidney. cDNA cloning and functional expression. J Biol Chem. (1993) 268:8418–21. 10.1016/S0021-9258(18)52891-X 8473283

[70] Lygate CA Neubauer S. The myocardial creatine kinase system in the normal, ischaemic and failing heart. In: Lopaschuk G Dhalla N, editors. Cardiac Energy Metabolism in Health and Disease. New York, NY: Springer (2014). 10.1007/978-1-4939-1227-8_10

[71] Neubauer S Remkes H Spindler M Horn M Wiesmann F Prestle J. Downregulation of the Na(+)-creatine cotransporter in failing human myocardium and in experimental heart failure. Circulation. (1999) 100:1847–50. 10.1161/01.CIR.100.18.1847 10545427

[72] Ten Hove M Chan S Lygate C Monfared M Boehm E Hulbert K. Mechanisms of creatine depletion in chronically failing rat heart. J Mol Cell Cardiol. (2005) 38:309–13. 10.1016/j.yjmcc.2004.11.016 15698837

[73] Walther T Tschope C Sterner-Kock A Westermann D Heringer-Walther S Riad A. Accelerated mitochondrial adenosine diphosphate/adenosine triphosphate transport improves hypertension-induced heart disease. Circulation. (2007) 115:333–44. 10.1161/CIRCULATIONAHA.106.643296 17210842

[74] Lygate CA Bohl S Ten Hove M Faller KM Ostrowski PJ Zervou S. Moderate elevation of intracellular creatine by targeting the creatine transporter protects mice from acute myocardial infarction. Cardiovasc Res. (2012) 96:466–75. 10.1093/cvr/cvs272 22915766 PMC3500046

[75] Wallis J Lygate CA Fischer A Ten Hove M Schneider JE Sebag-Montefiore L. Supranormal myocardial creatine and phosphocreatine concentrations lead to cardiac hypertrophy and heart failure: insights from creatine transporter-overexpressing transgenic mice. Circulation. (2005) 112:3131–9. 10.1161/CIRCULATIONAHA.105.572990 16286605

[76] Munzel T Camici GG Maack C Bonetti NR Fuster V Kovacic JC. Impact of oxidative stress on the heart and vasculature: part 2 of a 3-part series. J Am Coll Cardiol. (2017) 70:212–29. 10.1016/j.jacc.2017.05.035 28683969 PMC5663297

[77] Boveris A Chance B. The mitochondrial generation of hydrogen peroxide. General properties and effect of hyperbaric oxygen. Biochem J. (1973) 134:707–16. 10.1042/bj1340707 4749271 PMC1177867

[78] Blankenberg S Rupprecht HJ Bickel C Torzewski M Hafner G Tiret L. Glutathione peroxidase 1 activity and cardiovascular events in patients with coronary artery disease. N Engl J Med. (2003) 349:1605–13. 10.1056/NEJMoa030535 14573732

[79] Franco R Schoneveld OJ Pappa A Panayiotidis MI. The central role of glutathione in the pathophysiology of human diseases. Arch Physiol Biochem. (2007) 113:234–58. 10.1080/13813450701661198 18158646

[80] Yucel D Aydogdu S Cehreli S Saydam G Canatan H Senes M. Increased oxidative stress in dilated cardiomyopathic heart failure. Clin Chem. (1998) 44:148–54. 10.1093/clinchem/44.1.148 9550572

[81] Damy T Kirsch M Khouzami L Caramelle P Le Corvoisier P Roudot-Thoraval F. Glutathione deficiency in cardiac patients is related to the functional status and structural cardiac abnormalities. PLoS ONE. (2009) 4:e4871. 10.1371/journal.pone.0004871 19319187 PMC2655715

[82] Freeman LM Rush JE Milbury PE Blumberg JB. Antioxidant status and biomarkers of oxidative stress in dogs with congestive heart failure. J Vet Intern Med. (2005) 19:537–541. 10.1111/j.1939-1676.2005.tb02724.x 16095171

[83] Siliprandi N Ciman M Sartorelli L. Myocardial carnitine transport. Basic Res Cardiol. (1987) 82 (Suppl. 1):53–62. 10.1007/978-3-662-08390-1_7 3311009

[84] Vaz FM Wanders RJ. Carnitine biosynthesis in mammals. Biochem J. (2002) 361:417–29. 10.1042/bj3610417 11802770 PMC1222323

[85] Pierpont ME Judd D Goldenberg IF Ring WS Olivari MT Pierpont GL. Myocardial carnitine in end-stage congestive heart failure. Am J Cardiol. (1989) 64:56–60. 10.1016/0002-9149(89)90653-X 2662734

[86] Regitz V Shug AL Fleck E. Defective myocardial carnitine metabolism in congestive heart failure secondary to dilated cardiomyopathy and to coronary, hypertensive and valvular heart diseases. Am J Cardiol. (1990) 65:755–60. 10.1016/0002-9149(90)91383-H 2316456

[87] Martin MA Gomez MA Guillen F Bornstein B Campos Y Rubio JC. Myocardial carnitine and carnitine palmitoyltransferase deficiencies in patients with severe heart failure. Biochim Biophys Acta. (2000) 1502:330–6. 10.1016/S0925-4439(00)00061-2 11068176

[88] Keene BW Panciera DP Atkins CE Regitz V Schmidt MJ Shug AL. Myocardial L-carnitine deficiency in a family of dogs with dilated cardiomyopathy. J Am Vet Med Assoc. (1991) 198:647–50. 2019534

[89] Pierpont ME Foker JE Pierpont GL. Myocardial carnitine metabolism in congestive heart failure induced by incessant tachycardia. Basic Res Cardiol. (1993) 88:362–70. 8240228 10.1007/BF00800642

[90] Freeman LM. Interventional nutrition for cardiac disease. Clin Tech Small Anim Pract. (1998) 13:232–7. 10.1016/S1096-2867(98)80008-X 9842116

[91] Sanderson SL. Taurine and carnitine in canine cardiomyopathy. Vet Clin North Am Small Anim Pract. (2006) 36:1325–43, vii–viii. 10.1016/j.cvsm.2006.08.010 17085238

[92] Shekhawat PS Matern D Strauss AW. Fetal fatty acid oxidation disorders, their effect on maternal health and neonatal outcome: impact of expanded newborn screening on their diagnosis and management. Pediatr Res. (2005) 57:78R–86R. 10.1203/01.PDR.0000159631.63843.3E 15817498 PMC3582391

[93] Adams SH Hoppel CL Lok KH Zhao L Wong SW Minkler PE. Plasma acylcarnitine profiles suggest incomplete long-chain fatty acid beta-oxidation and altered tricarboxylic acid cycle activity in type 2 diabetic African-American women. J Nutr. (2009) 139:1073–81. 10.3945/jn.108.103754 19369366 PMC2714383

[94] Cheng ML Wang CH Shiao MS Liu MH Huang YY Huang CY. Metabolic disturbances identified in plasma are associated with outcomes in patients with heart failure: diagnostic and prognostic value of metabolomics. J Am Coll Cardiol. (2015) 65:1509–20. 10.1016/j.jacc.2015.02.018 25881932

[95] Hunter WG Kelly JP Mcgarrah RW 3rd Khouri MG Craig D Haynes C. Metabolomic profiling identifies novel circulating biomarkers of mitochondrial dysfunction differentially elevated in heart failure with preserved versus reduced ejection fraction: evidence for shared metabolic impairments in clinical heart failure. J Am Heart Assoc. (2016) 5:e003190. 10.1161/JAHA.115.003190 27473038 PMC5015273

[96] Chen WS Liu MH Cheng ML Wang CH. Decreases in circulating concentrations of short-chain acylcarnitines are associated with systolic function improvement after decompensated heart failure. Int Heart J. (2020) 61:1014–21. 10.1536/ihj.20-053 32879261

[97] Wanders RJ Komen J Kemp S. Fatty acid omega-oxidation as a rescue pathway for fatty acid oxidation disorders in humans. FEBS J. (2011) 278:182–94. 10.1111/j.1742-4658.2010.07947.x 21156023

[98] Li Q Laflamme DP Bauer JE. Serum untargeted metabolomic changes in response to diet intervention in dogs with preclinical myxomatous mitral valve disease. PLoS ONE. (2020) 15:e0234404. 10.1371/journal.pone.0234404 32555688 PMC7302913

[99] Abderhalden E. Experiment on the feeding with completely degraded nutrition substances. Z Physiol Chem. (1912) 77:22–58. 10.1515/bchm2.1912.77.1.22

[100] Young VR. Adult amino acid requirements: the case for a major revision in current recommendations. J Nutr. (1994) 124:1517S–23S. 10.1093/jn/124.suppl_8.1517S 8064412

[101] Bender DA. Biochemistry of tryptophan in health and disease. Mol Aspects Med. (1983) 6:101–97. 10.1016/0098-2997(83)90005-5 6371429

[102] Yano JM Yu K Donaldson GP Shastri GG Ann P Ma L. Indigenous bacteria from the gut microbiota regulate host serotonin biosynthesis. Cell. (2015) 161:264–76. 10.1016/j.cell.2015.02.047 25860609 PMC4393509

[103] Ghiboub M Verburgt CM Sovran B Benninga MA De Jonge WJ Van Limbergen JE. Nutritional therapy to modulate tryptophan metabolism and aryl hydrocarbon-receptor signaling activation in human diseases. Nutrients. (2020) 12:2846. 10.3390/nu12092846 32957545 PMC7551725

[104] Wirleitner B Rudzite V Neurauter G Murr C Kalnins U Erglis A. Immune activation and degradation of tryptophan in coronary heart disease. Eur J Clin Invest. (2003) 33:550–4. 10.1046/j.1365-2362.2003.01186.x 12814390

[105] Mangge H Stelzer I Reininghaus EZ Weghuber D Postolache TT Fuchs D. Disturbed tryptophan metabolism in cardiovascular disease. Curr Med Chem. (2014) 21:1931–7. 10.2174/0929867321666140304105526 24606499 PMC4922792

[106] Murr C Grammer TB Kleber ME Meinitzer A Marz W Fuchs D. Low serum tryptophan predicts higher mortality in cardiovascular disease. Eur J Clin Invest. (2015) 45:247–54. 10.1111/eci.12402 25586781

[107] Zuo H Ueland PM Ulvik A Eussen SJ Vollset SE Nygard O. Plasma biomarkers of inflammation, the kynurenine pathway, and risks of all-cause, cancer, and cardiovascular disease mortality: the hordaland health study. Am J Epidemiol. (2016) 183:249–58. 10.1093/aje/kwv242 26823439 PMC4753283

[108] Dschietzig TB Kellner KH Sasse K Boschann F Klusener R Ruppert J. Plasma kynurenine predicts severity and complications of heart failure and associates with established biochemical and clinical markers of disease. Kidney Blood Press Res. (2019) 44:765–76. 10.1159/000501483 31387104

[109] Magni G Amici A Emanuelli M Raffaelli N Ruggieri S. Enzymology of NAD+ synthesis. Adv Enzymol Relat Areas Mol Biol. (1999) 73:135–82, xi. 10.1002/9780470123195.ch5 10218108

[110] Okuda S Nishiyama N Saito H Katsuki H. 3-Hydroxykynurenine, an endogenous oxidative stress generator, causes neuronal cell death with apoptotic features and region selectivity. J Neurochem. (1998) 70:299–307. 10.1046/j.1471-4159.1998.70010299.x 9422375

[111] Perez-De La Cruz V Carrillo-Mora P Santamaria A. Quinolinic acid, an endogenous molecule combining excitotoxicity, oxidative stress and other toxic mechanisms. Int J Tryptophan Res. (2012) 5:1–8. 10.4137/IJTR.S8158 22408367 PMC3296489

[112] Reyes-Ocampo J Ramirez-Ortega D Cervantes GI Pineda B Balderas PM Gonzalez-Esquivel D. Mitochondrial dysfunction related to cell damage induced by 3-hydroxykynurenine and 3-hydroxyanthranilic acid: non-dependent-effect of early reactive oxygen species production. Neurotoxicology. (2015) 50:81–91. 10.1016/j.neuro.2015.08.003 26254737

[113] Ni W Watts SW. 5-hydroxytryptamine in the cardiovascular system: focus on the serotonin transporter (SERT). Clin Exp Pharmacol Physiol. (2006) 33:575–83. 10.1111/j.1440-1681.2006.04410.x 16789923

[114] Gustafsson BI Tommeras K Nordrum I Loennechen JP Brunsvik A Solligard E. Long-term serotonin administration induces heart valve disease in rats. Circulation. (2005) 111:1517–22. 10.1161/01.CIR.0000159356.42064.48 15781732

[115] Lancellotti P Nchimi A Hego A Dulgheru R Delvenne P Drion P. High-dose oral intake of serotonin induces valvular heart disease in rabbits. Int J Cardiol. (2015) 197:72–5. 10.1016/j.ijcard.2015.06.035 26114494

[116] Goldberg E Grau JB Fortier JH Salvati E Levy RJ Ferrari G. Serotonin and catecholamines in the development and progression of heart valve diseases. Cardiovasc Res. (2017) 113:849–57. 10.1093/cvr/cvx092 28863437 PMC5790145

[117] Fitzgerald LW Burn TC Brown BS Patterson JP Corjay MH Valentine PA. Possible role of valvular serotonin 5-HT(2B) receptors in the cardiopathy associated with fenfluramine. Mol Pharmacol. (2000) 57:75–81. 10617681

[118] Cremer SE Moesgaard SG Rasmussen CE Zois NE Falk T Reimann MJ. Alpha-smooth muscle actin and serotonin receptors 2A and 2B in dogs with myxomatous mitral valve disease. Res Vet Sci. (2015) 100:197–206. 10.1016/j.rvsc.2015.03.020 25843893

[119] Fung TC Vuong HE Luna CDG Pronovost GN Aleksandrova AA Riley NG. Intestinal serotonin and fluoxetine exposure modulate bacterial colonization in the gut. Nat Microbiol. (2019) 4:2064–73. 10.1038/s41564-019-0540-4 31477894 PMC6879823

[120] Li Q Larouche-Lebel E Loughran KA Huh TP Suchodolski JS Oyama MA. Gut dysbiosis and its associations with gut microbiota-derived metabolites in dogs with myxomatous mitral valve disease. mSystems. (2021) 6:e00111–21. 10.1128/mSystems.00111-21 33879495 PMC8546968

[121] Kato T Niizuma S Inuzuka Y Kawashima T Okuda J Tamaki Y. Analysis of metabolic remodeling in compensated left ventricular hypertrophy and heart failure. Circ Heart Fail. (2010) 3:420–30. 10.1161/CIRCHEARTFAILURE.109.888479 20176713

[122] Sansbury BE Demartino AM Xie Z Brooks AC Brainard RE Watson LJ. Metabolomic analysis of pressure-overloaded and infarcted mouse hearts. Circ Heart Fail. (2014) 7:634–42. 10.1161/CIRCHEARTFAILURE.114.001151 24762972 PMC4102656

[123] Schaffer SW Jong CJ Ramila KC Azuma J. Physiological roles of taurine in heart and muscle. J Biomed Sci. (2010) 17 (Suppl. 1):S2. 10.1186/1423-0127-17-S1-S2 20804594 PMC2994395

[124] Gaull GE Pasantes-Morales H Wright CE. Taurine in human nutrition: overview. Prog Clin Biol Res. (1985) 179:3–21. 3903756

[125] Gaull GE. Taurine as a conditionally essential nutrient in man. J Am Coll Nutr. (1986) 5:121–5. 10.1080/07315724.1986.10720119 3088081

[126] Pion PD Kittleson MD Rogers QR Morris JG. Myocardial failure in cats associated with low plasma taurine: a reversible cardiomyopathy. Science. (1987) 237:764–8. 10.1126/science.3616607 3616607

[127] Pion PD Kittleson MD Rogers QR Morris JG. Taurine deficiency myocardial failure in the domestic cat. Prog Clin Biol Res. (1990) 351:423–30. 2236148

[128] Qaradakhi T Gadanec LK Mcsweeney KR Abraham JR Apostolopoulos V Zulli A. The anti-inflammatory effect of taurine on cardiovascular disease. Nutrients. (2020) 12:2847. 10.3390/nu12092847 32957558 PMC7551180

[129] Schaffer S Jong CJ Shetewy A Ramila KC Ito T. Impaired energy production contributes to development of failure in taurine deficient heart. Adv Exp Med Biol. (2017) 975 (Pt. 1):435–46. 10.1007/978-94-024-1079-2_35 28849473

[130] Hayes KC Trautwein EA. Taurine deficiency syndrome in cats. Vet Clin North Am Small Anim Pract. (1989) 19:403–13. 10.1016/S0195-5616(89)50052-4 2658282

[131] Freeman LM Rush JE Brown DJ Roudebush P. Relationship between circulating and dietary taurine concentrations in dogs with dilated cardiomyopathy. Vet Ther. (2001) 2:370–8. 19746660

[132] Freid KJ Freeman LM Rush JE Cunningham SM Davis MS Karlin ET. Retrospective study of dilated cardiomyopathy in dogs. J Vet Intern Med. (2021) 35:58–67. 10.1111/jvim.15972 33345431 PMC7848368

[133] Sender R Fuchs S Milo R. Revised estimates for the number of human and bacteria cells in the body. PLoS Biol. (2016) 14:e1002533. 10.1371/journal.pbio.1002533 27541692 PMC4991899

[134] Qin J Li R Raes J Arumugam M Burgdorf KS Manichanh C. A human gut microbial gene catalogue established by metagenomic sequencing. Nature. (2010) 464:59–65. 10.1038/nature08821 20203603 PMC3779803

[135] Qin J Li Y Cai Z Li S Zhu J Zhang F. A metagenome-wide association study of gut microbiota in type 2 diabetes. Nature. (2012) 490:55–60. 10.1038/nature11450 23023125

[136] Turnbaugh PJ Ley RE Hamady M Fraser-Liggett CM Knight R Gordon JI. The human microbiome project. Nature. (2007) 449:804–10. 10.1038/nature06244 17943116 PMC3709439

[137] Tang WH Hazen SL. The contributory role of gut microbiota in cardiovascular disease. J Clin Invest. (2014) 124:4204–11. 10.1172/JCI72331 25271725 PMC4215189

[138] Nagatomo Y Tang WH. Intersections between microbiome and heart failure: revisiting the gut hypothesis. J Card Fail. (2015) 21:973–80. 10.1016/j.cardfail.2015.09.017 26435097 PMC4666782

[139] Tang WHW Li DY Hazen SL. Dietary metabolism, the gut microbiome, heart failure. Nat Rev Cardiol. (2019) 16:137–54. 10.1038/s41569-018-0108-7 30410105 PMC6377322

[140] Von Eckardstein A. Trimethyllysine and trimethylamine-N-oxide - pathogenic factors or surrogate markers of increased cardiovascular disease risk? J Intern Med. (2020) 288:484–6. 10.1111/joim.13086 32424985

[141] Seo J Matthewman L Xia D Wilshaw J Chang YM Connolly DJ. The gut microbiome in dogs with congestive heart failure: a pilot study. Sci Rep. (2020) 10:13777. 10.1038/s41598-020-70826-0 32792610 PMC7426839

[142] Rath S Heidrich B Pieper DH Vital M. Uncovering the trimethylamine-producing bacteria of the human gut microbiota. Microbiome. (2017) 5:54. 10.1186/s40168-017-0271-9 28506279 PMC5433236

[143] Bianchi VE. Impact of nutrition on cardiovascular function. Curr Probl Cardiol. (2020) 45:100391. 10.1016/j.cpcardiol.2018.08.003 30318107

[144] Kernan WN Ovbiagele B Black HR Bravata DM Chimowitz MI Ezekowitz MD. Guidelines for the prevention of stroke in patients with stroke and transient ischemic attack: a guideline for healthcare professionals from the American heart association/American stroke association. Stroke. (2014) 45:2160–236. 10.1161/STR.0000000000000024 24788967

[145] Taylor RS Ashton KE Moxham T Hooper L Ebrahim S. Reduced dietary salt for the prevention of cardiovascular disease: a meta-analysis of randomized controlled trials (Cochrane review). Am J Hypertens. (2011) 24:843–53. 10.1038/ajh.2011.115 21731062

[146] Alderman MH Cohen HW. Dietary sodium intake and cardiovascular mortality: controversy resolved? Curr Hypertens Rep. (2012) 14:193–201. 10.1007/s11906-012-0275-6 22639013

[147] Freeman LM Rush JE Kehayias JJ Ross J. N. Jr. Meydani SN. Nutritional alterations and the effect of fish oil supplementation in dogs with heart failure. J Vet Intern Med. (1998) 12:440–8. 10.1111/j.1939-1676.1998.tb02148.x 9857337

[148] Freeman LM Rush JE. Nutrition and cardiomyopathy: lessons from spontaneous animal models. Curr Heart Fail Rep. (2007) 4:84–90. 10.1007/s11897-007-0005-6 17521500

[149] Freeman LM. Beneficial effects of omega-3 fatty acids in cardiovascular disease. J Small Anim Pract. (2010) 51:462–70. 10.1111/j.1748-5827.2010.00968.x 20673293

[150] Billingsley HE Hummel SL Carbone S. The role of diet and nutrition in heart failure: a state-of-the-art narrative review. Prog Cardiovasc Dis. (2020) 63:538–51. 10.1016/j.pcad.2020.08.004 32798501 PMC7686142

[151] Rush JE Freeman LM Brown DJ Brewer BP Ross J. N. Jr.. Clinical, echocardiographic, and neurohormonal effects of a sodium-restricted diet in dogs with heart failure. J Vet Intern Med. (2000) 14:513–20. 10.1111/j.1939-1676.2000.tb02269.x 11012115

[152] Suematsu N Ojaimi C Recchia FA Wang Z Skayian Y Xu X. Potential mechanisms of low-sodium diet-induced cardiac disease: superoxide-NO in the heart. Circ Res. (2010) 106:593–600. 10.1161/CIRCRESAHA.109.208397 20007914 PMC2828877

[153] Freeman LM Rush JE Markwell PJ. Effects of dietary modification in dogs with early chronic valvular disease. J Vet Intern Med. (2006) 20:1116–26. 10.1111/j.1939-1676.2006.tb00709.x 17063703

[154] Mcgarrah RW Crown SB Zhang GF Shah SH Newgard CB. Cardiovascular metabolomics. Circ Res. (2018) 122:1238–58. 10.1161/CIRCRESAHA.117.311002 29700070 PMC6029726

[155] Kondreddy VK Anikisetty M Naidu KA. Medium-chain triglycerides and monounsaturated fatty acids potentiate the beneficial effects of fish oil on selected cardiovascular risk factors in rats. J Nutr Biochem. (2016) 28:91–102. 10.1016/j.jnutbio.2015.10.005 26878786

[156] Huwait EA. Combination of vitamin E and L-carnitine is superior in protection against isoproterenol-induced cardiac affection: a histopathological evidence. Folia Morphol. (2018) 78:274–82. 10.5603/FM.a2018.0070 30106462

